# Are Adolescent Climbers Aware of the Most Common Youth Climbing Injury and Safe Training Practices?

**DOI:** 10.3390/ijerph17030812

**Published:** 2020-01-28

**Authors:** Rachel N. Meyers, Steven L. Hobbs, David R. Howell, Aaron J. Provance

**Affiliations:** 1 Physical Therapy Division, Duke University School of Medicine, Durham, NC 27710, USA; 2 Department of Integrative Physiology, University of Colorado Boulder, Boulder, CO 80309, USA; steven.hobbs@colorado.edu; 3 Sports Medicine Center, Children’s Hospital Colorado, Aurora, CO 80045, USA; David.howell@childrenscolorado.org (D.R.H.); aaron.provance@childrenscolorado.org (A.J.P.); 4 School of Medicine, University of Colorado Hospital, Aurora, CO 80045, USA

**Keywords:** rock climbing, epiphyseal fracture, growth plate injury, finger injury, youth, perceptions

## Abstract

Finger growth plate injuries are the most common youth climbing injuries. The purpose of our study was to understand youth awareness of the most common youth climbing injury and safe training practices. We surveyed climbers, ages eight to 18 years old, at the 2017 USA Climbing Sport and Speed Youth National Championships. A total of 267 climbers completed the survey (mean age = 14 ± 2.7 years; 52% male). The A2 pulley injury was reported as the most common youth climbing injury by the largest portion of participants, 36%. The second most commonly identified injury was at the growth plate of the finger, 15% of participants, which was reported as significantly less than the A2 pulley injury, *p* < 0.001. Six percent of climbers reported the correct safe age to start double dyno campus board training. Roughly 18% of athletes identified growth plate injuries exclusively as a stress fracture, whereas 29.2% of those climbers self-reported as informed about finger growth plate injuries, but only 7.4% of climbers who self-reported as uninformed answered this question correctly. Misperceptions about skeletally-immature climbing injuries are prevalent amongst youth climbers. Education on the prevalence of finger growth plate injuries and the scarcity of A2 pulley injuries in youth climbers can increase diagnostic accuracy, improve care, and reduce long-term complications.

## 1. Introduction

In the United States, it is estimated that 60 million children aged six to 18 years participate in some form of organized sport [1]. One of the many inherent risks in youth sports is overuse injury, as an estimated 50% of injuries seen in pediatric sports medicine clinics are overuse in nature [2]. In the long term, overuse injury has been associated with an increased risk of osteoarthritis [3,4] and potential complications to musculoskeletal development [4,5]. However, there is currently a dearth of literature examining the education of youth athletes about common pediatric sports injuries. In order to reduce the risk for future overuse injury, it is essential to understand injury awareness and risk factors from the youth athlete’s perspective.

Competition climbing is one of the many sports at risk for overuse injury. Repetitive overuse from bouldering and sport climbing, two subdisciplines in climbing, had the highest incidence of injury at 1.13 injuries per 1000 h as compared to acute mechanisms of injury, such as a fall or strenuous move [6]. In 2012, roughly 1.5 million youth between the ages of six and seventeen participated in bouldering or sport climbing in the United States [7], with that number estimated to rise following the inclusion of climbing in the Tokyo 2020 Olympic Games. Similar to gymnastics, competitive climbers often reach elite levels at a young age, prior to skeletal maturation, which leads to an increased concern for physeal injury. Unlike many other sports, competition climbing is associated with overuse injuries that are almost exclusive to climbing. Consequently, climbing-specific injuries, particularly in youth, are occurring at rates that appear to be outpacing education and awareness about these injuries. 

The most common injury in adolescent rock climbers is a repetitive stress epiphyseal fracture of the finger [8,9,10,11], also known as a growth plate injury to the finger, but large-scale incidence studies have yet to determine the prevalence of this injury among adolescents. A recent study [11] reported that 70% of finger injuries, up to age fourteen, were diagnosed as an epiphyseal fracture, as these injuries most often occur around pubescent age [8,9,10]. A previous known risk for epiphyseal stress fractures in youth climbing is excess use of the crimp grip [12]. In a recent study, 64.3% out of 22 epiphyseal fractures in youth climbers were caused from the crimp grip, and this hand hold was the preferred grip in 71.4% of climbers with this injury [12]. Furthermore, according to the British Mountain Council, in cooperation with the International Mountaineering Association Medical Commission (UIAA MedCom), adolescent climbers younger than eighteen years of age are advised to avoid a climbing-specific training technique, known as double dyno campus boarding, which can damage the physis [11]. Double dyno campus boarding is similar to the crimp grip, as it causes simultaneous flexion of the proximal interphalangeal (PIP) joints and extension of the distal interphalangeal (DIP) joints. Double dyno campus boarding requires repetitive and dynamic movements on a series of holds exclusively with the upper body. Amongst junior competition climbers, the majority who trained regularly on the campus board developed finger growth plate injuries [13]. Previous research has yet to examine if youth climbers are aware of the safe age to start participating in double dyno campus boarding.

Unlike finger growth plate injuries, the A2 pulley rupture is the most common climbing injury in skeletally-mature athletes (i.e., 49%) [11] and is rare in skeletally-immature athletes [14]. The A2 pulley injury is more often reported and receives more attention, as a body of literature on A2 pulley injuries existed for more than ten years prior to the first report of finger growth plate injuries in adolescent climbers [15,16,17]. A2 pulley ruptures often have a “popping” sound that is distinct to this injury [18], commonly caused by simultaneous flexion of the PIP joint and extension of the DIP joint.

A paucity of literature exists examining athlete awareness about youth sports injuries, and none exists specific to youth climbing. Unspecific to climbing, previous literature in adults has suggested that elite athletes significantly underestimated the disruptive effects of their injury [19]. Furthermore, one study [20] found a dramatic underreporting of concussions in high school athletes, an often mainstreamed topic in sports medicine, due to lack of knowledge, failure to recognize symptoms, and failure to receive medical attention. Similar to these findings and specific to climbing, finger growth plate injuries in youth climbers are also likely to go unreported, especially among those who climb grades identical to elite skeletally-mature climbers [10]. Growth arrest, long-term pathological changes, and decreased range of motion can result in climbers who delay reporting joint pain or ignore medical advice [10]. As the number of adolescent competition climbers increases, improved education of athletes, coaches, and medical professionals about finger growth plate injuries in youth climbers could help reduce injury rates.

On the basis of the existing gaps in the literature, the purpose of our study was to examine the awareness and knowledge of youth-specific climbing injuries and safe training practices from the perspective of adolescent climbers. Our hypotheses are as follows: (1) The majority of elite adolescent rock climbers believe the most common pediatric climbing injury is the A2 pulley rupture, and (2) the majority of elite adolescent rock climbers are uninformed about safe training techniques among skeletally-immature rock climbers.

## 2. Methods

### 2.1. Participants

Study participants included elite adolescent rock climbers, ages 8 to 18 years, competing in the 2017 USA Climbing Youth Sport and Speed (SCS) National Championships. The study was approved by the University of Colorado Boulder Institutional Review Board and the athletes and parents of athletes under 18 years of age provided written informed assent and consent, respectively. The SCS National Championship competitors included in this study competed in the disciplines of sport climbing, speed climbing, or both.

### 2.2. Study Design

Elite adolescent rock climbers completed study questionnaires during athlete registration at the 2017 USA Climbing Youth Sport and Speed National Championships. Inclusion criteria were athletes ages 8 to18 years competing in the 2017 USA Climbing SCS National Championships. Subjects answered multiple-choice questions to the best of their ability about their awareness of the most common youth climbing injury and safe training practices. Survey questions consisted of the following four types of questions: (1) demographic, training, and injury questions; (2) injury ranking questions; (3) injury (factual) knowledge; and (4) safe training practices, all of which are described in more detail in Section 2.3.

### 2.3. Questionnaire

#### 2.3.1. Demographic, Training, and Injury Questions

Demographic, training, and injury questions included age, gender, years climbing, average hours climbing per week, average number of rest days per week, and most difficult climbing grade completed in the past 3 months, 6 months, and 12 months. Additional questions asked the percentage of time spent climbing indoors vs. outdoors; the percentage of time spent throughout the year bouldering, sport climbing, and speed climbing; and the number of competitions they competed in per year. We also asked participants what injuries they had sustained from climbing or training for climbing. Participants could choose from the following: pulley injury to finger, growth plate injury to finger, rotator cuff/labrum injury of shoulder, back/posture pain, ankle sprain, elbow tendonitis, meniscus tear of knee, tendon injury to wrist, other, or none. In our survey, these categorical questions were used to better determine which injuries were sustained by youth climbers.

#### 2.3.2. Injury Ranking Questions

Participants were asked to rank on a scale from 1 (most common) to 8 (least common) what they believe the most common youth climbing injury is from a list of injuries including pulley injury to finger, growth plate injury to finger, rotator cuff/labrum injury of shoulder, back/posture pain, ankle sprain, elbow tendonitis, meniscus tear of knee, and tendon injury to wrist.

#### 2.3.3. Injury (Factual) Knowledge

Participants were asked if they had been informed of growth plate injuries to the finger, as well as if they had been informed of A2 pulley injuries. Participants were asked to further define finger growth plate injuries by selecting “yes,” “no”, or “I don’t know” options for each of the following injury types: tendon injury, a ligament injury, stress fracture, dislocation, and a kind of pulley injury. If an athlete answered that a growth plate injury to the finger is exclusively a stress fracture, we defined that participant as “GPI informed.” All other participants were defined as “GPI uninformed.”

#### 2.3.4. Safe Training Practices

Lastly, participants were asked questions based on their awareness of safe training practices in youth rock climbers. The participants were asked what age they believe is safe to start double dyno campus board training and answers were constrained to 8 and older, 10 and older, 13 and older, 16 and older, 18 and older, “age doesn’t matter,” or “I have no idea.” Additionally, we asked participants if they train with additional weights or if they participate in campus board training.

### 2.4. Statistical Analysis

Chi-square tests were used as omnibus tests for multiple comparisons of percentages based on count data. When omnibus tests were significant, separate Chi-square tests were used for pairwise comparisons using an equality of proportions tests, and *p*-values adjusted using the Bonferroni correction. Means of quantitative variables are reported with standard deviations (SD), 95% confidence intervals (CI) are presented for percentages using the Clopper–Pearson method [21], but these were not used for hypothesis testing. Statistical analyses were performed in R (version 3.6.2) (R Foundation for Statistical Computing, Vienna, Austria).

## 3. Results

### 3.1. Education and Awareness

#### 3.1.1. Questionnaire

##### Demographic, Training, and Injury Questions

Among the 613 total competitors, 267 (43.6%) completed the survey (mean age =14.0 ± 2.7 SD, 51.9% male and 48.1% female). When asked what injuries an athlete had sustained from climbing, 41.8% of athletes reported never having sustained an injury, 14.9% reported having sustained a pulley injury, and 4.6% reported having sustained a growth plate injury to the finger, the second least common answer (Table 1).

#### 3.1.2. Injury Ranking Questions

**Figure 1:**
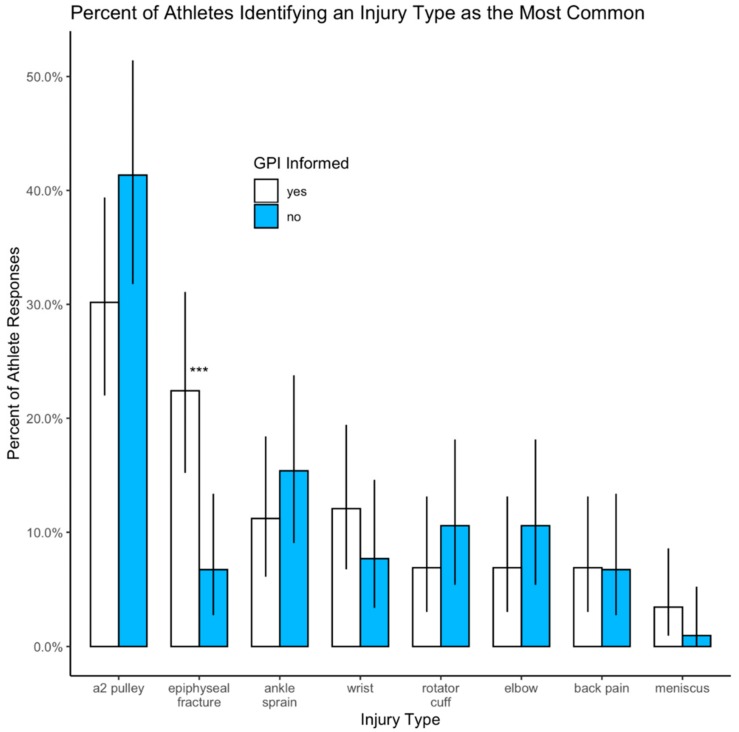
Identification of the most common injury type by GPI informed and GPI uninformed athletes. Significant differences between the percentage of GPI informed and GPI uninformed for an epiphyseal fracture are indicated with three asterisks (*p* < 0.001).

Slightly more than 35% of all athletes ranked the A2 pulley injury as the most common youth climbing injury. Chi-square tests revealed significant differences between the number of times each injury was reported as most common among all participants (χ^2^(7) = 124, *p* < 0.001), the self-reported GPI informed (χ^2^(7) = 54.6, *p* < 0.001), and the self-reported GPI uninformed (χ^2^(7) = 89.1, *p* < 0.001). The A2 pulley was selected as the most common injury type more than any other injury and significantly more than finger growth plate injuries for all athletes combined (χ^2^(1) = 23.3, *p* < 0.001) and for the GPI uninformed (Figure 1, χ^2^(1) = 35.3, *p* < 0.001), but not for the GPI informed (Figure 1, χ^2^(1) = 1.42, *p* = 0.23).

#### 3.1.3. Injury (Factual) Knowledge

**Figure 2:**
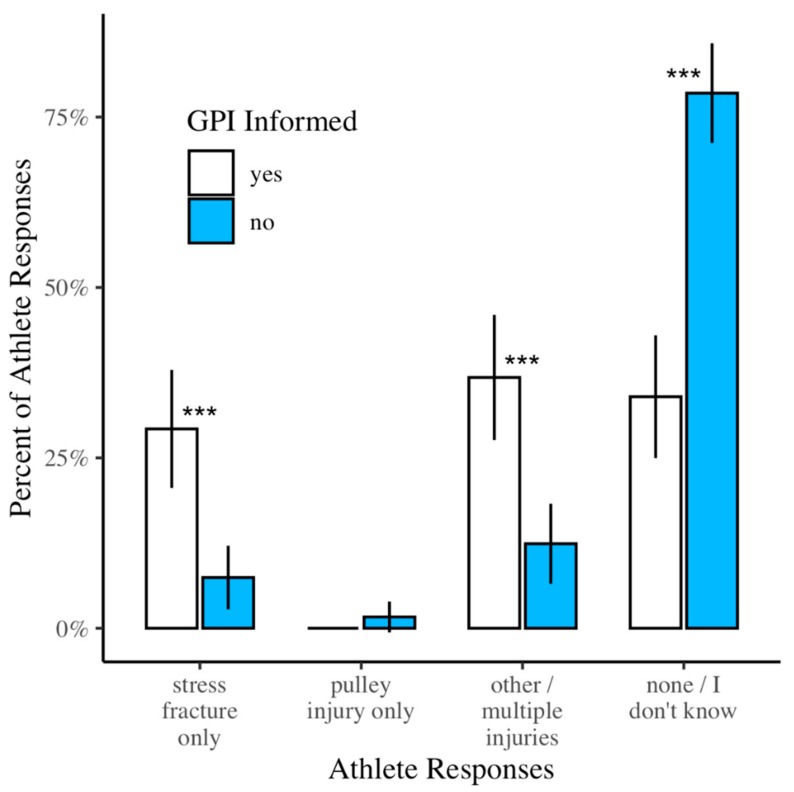
Athlete responses to the question “Growth plate injury to the finger is a…” Statistically significant comparisons between GPI informed and GPI uninformed are indicated by three asterisks (*p* < 0.001).

Almost half (48.9%) of athletes reported they were informed about growth plate injuries to the finger (GPI informed). Out of all study participants, 17.6% identified growth plate injuries exclusively as a stress fracture. Of the GPI informed and uninformed, 29.2% and 7.4% respectively, answered this question correctly and the difference between the two groups was statistically significant (Table 2 and Figure 2, χ^2^(1) = 18.3, *p* < 0.001). Of the GPI informed and uninformed, 0% and 1.6%, respectively, indicated growth plate injuries were A2 pulley injuries, but the difference was not statistically significant (Figure 2, χ^2^(1) = 0.382, *p* > 0.99). Among all athletes, 23.8% indicated that growth plate injuries were one or more of the other injury types listed (not a stress fracture or A2 pulley injury), while 36.8% and 12.4% of GPI informed and uninformed, respectively, answered this way and the difference was significant (Figure 2*,* χ^2^(1) = 17.2, *p* < 0.001.) Of all athletes, 57.7% reported that they specifically did not know the definition of a growth plate injury. Of the GPI informed and uninformed, 34.0% and 78.5%, respectively, answered “none/I don’t know” for this question and the difference was statistically significant (Figure 2, χ^2^(1) = 44.1, *p* < 0.001).

Among the 39 athletes who reported they sustained an A2 pulley injury, 82.1% self-reported as uninformed about growth plate injuries. Within the GPI uninformed, 66.4% were involved in two or more risk factors for growth plate injuries. Included risk factors were campus boarding and training with additional weights (i.e., weighted pull ups).

### 3.2. Safe Training Practices

**Figure 3:**
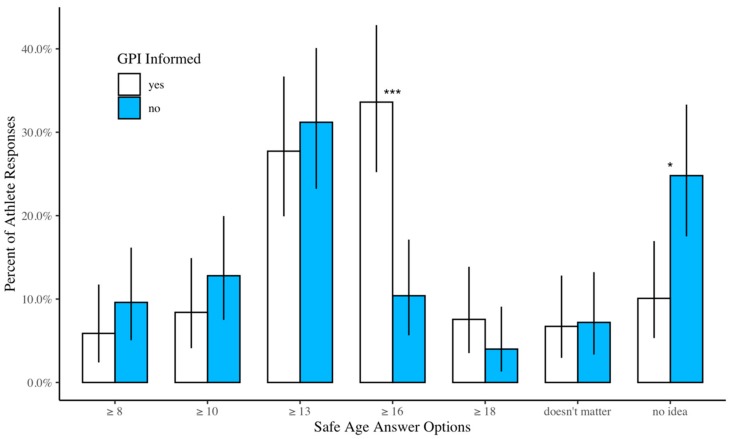
Athlete responses to the question “What is the safe age to start double dyno campus boarding?” Statistically significant comparisons between GPI informed and GPI uninformed are indicated with one asterisk (*p* < 0.05) or three asterisks (*p* < 0.001).

Among the athletes, 5.7% reported the safe age (18+) to start double dyno campus board training (Figure 3 and Table 2). The remaining 94.3% of athletes believed the safe age to start double dyno campus board training is less than eighteen, claimed age did not matter, or answered they did not know (Figure 3). The percentage of athletes correctly reporting the safe age to start double dyno campus board training was not significantly different between the GPI informed, i.e., 7.5% and GPI uninformed, i.e., 4% (χ^2^(1) = 0.848, *p* = 0.36) (Figure 3). Compared to the uninformed, the GPI informed were more likely to answer “16+” for the safe age to start double dyno campusing (32% vs. 11%, χ^2^(1) = 14.7, *p* < 0.001), but less likely to answer “no idea”. Lastly, out of 76.2% of athletes who trained on the campus board, 3.5% reported they had sustained a finger growth plate injury.

## 4. Discussion

Our data suggest widespread misperceptions exist about finger growth plate injuries and A2 pulley injuries among elite adolescent rock climbers. We found that among our athletes, the A2 pulley injury was reported three times (15%) more frequently than a growth plate injury to the finger (5%). This finding was not in agreement with previous literature [8,9,10,11] that found growth plate injuries to the finger to be the most common injury in youth climbers. Although pulley injuries are rare in skeletally-immature climbers, [14] we suspected more athletes to have reported an A2 pulley injury than a growth plate injury due to the high incidence of A2 pulley injuries being reported in skeletally-mature climbers [11]. Due to this high incidence, as well as the longer documented knowledge of the A2 pulley injury, the youth athletes in our study could have misperceptions regarding finger pain. Consequently, youth-specific climbing injuries have been less researched and reported, thus, lack of awareness of these growth plate injuries could have led to misperceptions and possible misdiagnoses.

Further supporting possible misdiagnoses, we found that 82% of athletes who reported they had a pulley injury were uninformed about growth plate injuries to the finger. A previous study [18] reported that climbers in the age group of our study were too young for an A2 pulley injury, indicating that growth plate injuries could have been present among these individuals. Skeletally-mature climbers suffering from A2 pulley pain have a mean age of 30.7 years, while those without A2 pulley pain are significantly younger at 22.6 years [18]. In our study, the mean age of 15 years for reported pulley injuries was highly comparable to the mean age for reported growth plate injuries found in other studies [8,9,10]. The mean age of 15 years is approximately half that of skeletally-mature climbers reported to experience A2 pulley pain. In light of these points, we suspect that some of the youth athletes in our study could have been misdiagnosed, either by a medical professional or self-diagnosis, as having an A2 pulley injury. Among the athletes in our study who were uninformed about growth plate injuries, 66% were involved in two or more risk factors for growth plate injuries. In addition, 47% of the athletes in our study were between the ages of 13 to 16 years, when growth plate injuries are more likely to occur [8,9,10]. These findings further support the need for education among athletes, coaches, and physicians about growth plate injuries in elite adolescent climbers.

Our study demonstrated some misperceptions about safe training practices for skeletally-immature athletes. Not only was the A2 pulley injury reported as the most common youth climbing injury, but the majority of athletes were also unaware of the safe age to start double dyno campus board training. According to the British Mountain Council, adolescent climbers younger than eighteen years of age should avoid double dyno campus board training as it can lead to injury of the physis. In our study, 5.7% of climbers correctly reported the safe age to start double dyno campus board training, and out of those who reported they were aware of growth plate injuries, 7.5% answered with the correct age of eighteen and older. The majority of athletes in our study reported that the safe age to start double dyno campus board training is thirteen and older. This is a potentially dangerous misconception, as it is the most vulnerable time for a growth plate injury [8,9,10]. Our findings do not directly suggest campus board training is a risk factor for epiphyseal fractures, as reported in previous literature [11,13,15]. This discrepancy could stem from a weakness in our study, which did not specifically ask athletes if they performed the double dyno movements on campus boards that have been shown to place youth athletes at an increased risk for growth plate injuries [8].

Only 3.5% out of 76.2% of athletes in our study who trained on the campus board reported they had sustained a growth plate injury. Previous literature found that close to two-thirds of adolescent climbers who train on the campus board developed an epiphyseal fracture [15]. It is possible that the youth climbers who reported that they had a pulley injury were actually suffering from a growth plate injury due to a lack of awareness of the commonality of this injury. In addition, finger injuries in elite youth climbers may go unreported, especially among those who climb grades identical to elite skeletally-mature climbers [10]. Educating adolescent climbers about the prevalence of GPIs and the scarcity of A2 pulley injuries in youth climbers could increase diagnostic accuracy, improve care, and reduce long-term damage or loss of function.

Although we did not find a high incidence of growth plate injuries from campus boarding, it is important to note the differences among study designs. The findings reported in a previous literature [15] was a case series, where all of the athletes were at prime age for physeal overuse injury. Due to our cross-sectional study design, we included athletes between the ages of eight to 18 years. Although all athletes in our study could train on the campus board, those who are likely to report a history of a finger growth plate injury are those who are either currently at pubertal age or older. Due to the retrospective nature of our survey, the younger prepubescent climbers are likely to not have sustained a growth plate injury, making our incidence rate lower than previous studies.

A limitation of our study is that all of our findings are from self-reported data and lacked validation by medical professionals or radiographic findings. Consequently, our self-reported injury data is likely affected by erroneous self-diagnoses and possibly erroneous diagnoses from medical professionals unfamiliar with youth-specific climbing injuries. However, self-reported data supported the main purpose of our study, which was to identify the awareness of injuries and safe training practices in the skeletally-immature climber. In addition, younger climbers may not be familiar with the terminology “stress fracture” or know the accurate definition of a stress fracture and could have asked a parent for help while filling out the survey. Consequently, this limitation would affect the accuracy of correctly identifying the athletes who were informed or uninformed about growth plate injuries in the fingers. We also did not examine athlete awareness of the crimp grip as a risk for physeal injury, and future studies should assess this knowledge. Finally, we only asked youth athletes their perceptions regarding injury and safe training practices, but did not include coaches’ and physicians’ perceptions. Improving education of injury mechanism and risk factors in youth climbers must be a team effort, and the awareness levels of physicians and coaches should be examined in future studies. Furthermore, our questionnaire needs to be validated if replicated in future studies.

Although youth climbers demonstrate misperceptions regarding injury and safe training practices, we recommend that climbing gyms put signs up next to the campus board indicating the safe age to start double dyno campus boarding. Additionally, coaches and physicians should be aware of the warning signs of a finger growth plate injury, such as pain on the dorsal aspect of the PIP joint, along with its known risk factors. Another suggestion to gain awareness is to require educational videos or online training for coaches regarding the signs and risk factors for a finger growth plate injury prior to the competition season.

Finally, our intent is not to discourage young athletes from climbing. Climbing provides many physical and mental benefits that largely outweigh the risks for injury. Our findings could help increase the awareness of adolescent climbing injuries, and therefore, athletes, coaches, and parents would better understand how to reduce the risk for growth plate injuries and potential long-term complications.

## 5. Conclusions

Elite youth climbers believed the most common climbing-specific injury among skeletally immature climbers is the A2 pulley injury. In addition, the majority of adolescent climbers in our study did not know the safe age to start double dyno campus board training. As climbing enters the Tokyo 2020 Olympic Games and continues to grow as a youth sport, addressing this lack of awareness could help athletes, parents, and coaches understand the risk for growth plate injuries and guide adolescent climbers and parents to seek medical attention when appropriate. Educating youth, coaches, and parents about finger injuries would first effect better diagnosis and treatment, and if safe training regimens and rules are adapted with general agreement, could then reduce the incidence of growth plate injuries in youth climbers.

## Figures and Tables

**Table 1:** Percentage of self-reported injuries by type.

Self-Reported Injuries by Type	Frequency (%)	Sex (% Male)
none	41.8%	59.6%
other	19.9%	36.5% ^1^
ankle sprain	16.1%	23.8%
pulley injury	14.9%	64.1%
back/posture pain	13.4%	45.7%
tendon injury to wrist	10.9%	42.9%
elbow tendonitis	10.0%	46.2%
rotator cuff/labrum injury of shoulder	9.2%	41.7%
growth plate injury to finger	4.6%	58.3%
meniscus tear of knee	1.1%	0.0%

**Table 2:** Percent and 95% CI of athlete responses for all athletes, growth plate injury (GPI) informed, and growth plate injury (GPI) uninformed.

		All Athletes		GPI Informed		GPI Uninformed	
		Percent	95% CI	Percent	95% CI	Percent	95% CI
Figure 1	A2 pulley most common	35.5%	(29.1, 42.2)	30.2%	(22.0, 39.4)	41.3%	(31.8, 51.4)
Figure 1	GPI most common	15.0%	(10.6, 20.4)	22.4%	(0.152, 0.311)	6.7%	(2.7, 13.4)
Figure 2	Identified GPI *exclusively* as a stress fracture	17.6%	(10.2, 19.8)	29.2%	(16.7, 33.8)	7.4%	(2.4, 11.6)
Figure 3	Correctly identified safe double dyno age	5.7%	(3.2, 9.4)	7.5%	(2.8, 12.2)	4.0%	(.56, 7.4)
